# The Extracellular Redox State Modulates Mitochondrial Function, Gluconeogenesis, and Glycogen Synthesis in Murine Hepatocytes

**DOI:** 10.1371/journal.pone.0122818

**Published:** 2015-03-27

**Authors:** Laura Nocito, Amber S. Kleckner, Elsia J. Yoo, Albert R. Jones IV, Marc Liesa, Barbara E. Corkey

**Affiliations:** Department of Medicine, Boston University, Boston, Massachusetts, United States of America; Instituto Nacional de Cardiologia, MEXICO

## Abstract

Circulating redox state changes, determined by the ratio of reduced/oxidized pairs of different metabolites, have been associated with metabolic diseases. However, the pathogenic contribution of these changes and whether they modulate normal tissue function is unclear. As alterations in hepatic gluconeogenesis and glycogen metabolism are hallmarks that characterize insulin resistance and type 2 diabetes, we tested whether imposed changes in the extracellular redox state could modulate these processes. Thus, primary hepatocytes were treated with different ratios of the following physiological extracellular redox couples: β-hydroxybutyrate (βOHB)/acetoacetate (Acoc), reduced glutathione (GSH)/oxidized glutathione (GSSG), and cysteine/cystine. Exposure to a more oxidized ratio via extracellular βOHB/Acoc, GSH/GSSG, and cysteine/cystine in hepatocytes from fed mice increased intracellular hydrogen peroxide without causing oxidative damage. On the other hand, addition of more reduced ratios of extracellular βOHB/Acoc led to increased NAD(P)H and maximal mitochondrial respiratory capacity in hepatocytes. Greater βOHB/Acoc ratios were also associated with decreased β-oxidation, as expected with enhanced lipogenesis. In hepatocytes from fasted mice, a more extracellular reduced state of βOHB/Acoc led to increased alanine-stimulated gluconeogenesis and enhanced glycogen synthesis capacity from added glucose. Thus, we demonstrated for the first time that the extracellular redox state regulates the major metabolic functions of the liver and involves changes in intracellular NADH, hydrogen peroxide, and mitochondrial respiration. Because redox state in the blood can be communicated to all metabolically sensitive tissues, this work confirms the hypothesis that circulating redox state may be an important regulator of whole body metabolism and contribute to alterations associated with metabolic diseases.

## Introduction

Reduction/oxidation (“redox”) reactions involve the transfer of electrons between molecules, in which the reduced form of a molecule is oxidized after electron(s) are transferred to another molecule. In this regard, the reduced and oxidized forms of the same molecule are named *redox couples* and their inter-conversion usually requires a second redox couple, which also provides and accepts electrons. For instance, lactate (Lac) and pyruvate (Pyr) form a redox couple and their inter-conversion requires the NADH/NAD^+^ redox pair to mediate the transfer of electrons. These processes of electron transfer are involved in hundreds of vital reactions, can be catalyzed by enzymes, and a common byproduct of these reactions is the generation of reactive oxygen species (ROS). ROS production in excess can be deleterious for the cell, as it can cause oxidative damage to cellular lipids, proteins, and nucleic acids. However, molecules of ROS also act as essential signaling molecules through different mechanisms. Thus, it is not surprising that redox regulation and ROS mediation are multifaceted and differ among tissues and cellular compartments [1,2]. Despite the large number of redox pairs found inside the cell and their complex regulation, the NADH/NAD^+^ redox state can be estimated in the cytosol by measuring the Lac/Pyr ratio and inside the mitochondrial matrix by measuring β-hydroxybutyrate (βOHB)/acetoacetate (Acoc) ratios (review: [3]). The Lac/Pyr ratio is typically maintained near 10, while the βOHB/Acoc ratio is typically around 1 [3]. Similarly, reduced/oxidized glutathione (GSH/GSSG), which is often reported as the cell potential, is maintained around -280 mV in the mitochondria and about -260 mV to -200 mV in the cytosol [1]. The cysteine/cystine couple is not in equilibrium with the glutathione couple, and is typically more oxidized in all compartments [4].

Because of the abundance of redox reactions required for the production of ATP using molecular oxygen, mitochondria can be major sites of redox reactions and ROS production inside the cell. As such, they contain a much greater concentration of total NAD(P)H and the ratio of pyridine nucleotide redox potential is much more reduced than the cytosol [1]. A paradigmatic example illustrating the importance of mitochondrial redox state for tissue function is the liver. Mitochondrial function is essential for liver metabolism both in the fed and fasted states. During fasting, the main metabolic function of the liver is to produce glucose (by glycogenolysis and gluconeogenesis) and ketone bodies (through mitochondrial fatty acid β-oxidation) that serve as fuel to other tissues. These processes are tightly linked to mitochondrial function and increased availability of mitochondrial NADH enhances them. On the other hand, during the fed state, glucose can enter the liver and is converted to glycogen. Also during the fed state, glucose is catabolized to produce pyruvate by glycolysis, which is then imported to the mitochondria and used to synthesize citrate, simultaneously generating NADH. These reactions contribute to hepatic lipogenesis for lipoprotein formation and indeed this process can decrease normal mitochondrial fatty acid β-oxidation, despite the fact that hepatocytes mostly use fatty acid oxidation to produce NADH and ATP in the mitochondria (review: [5]).

Understanding the roles of these distinct yet interactive redox regulation systems will better direct therapeutics for aging and redox-related conditions. Indeed, deregulation of intracellular redox control has been implicated in multiple diseases, particularly the etiology of obesity [6] and complications of type 2 diabetes [7]. In this regard, consequences of extracellular changes are less understood in regard to effects on normal tissue metabolism and, particularly, why changes in the circulating (bloodstream) redox state are associated with different physiological and pathogenic conditions (e.g. [8,9]). As the circulating redox state is mostly constituted by release of intracellular metabolites, we recently proposed that changes in the extracellular redox state are used as signals to coordinate and integrate the metabolism of multiple tissues (i.e. liver, muscle, adipocytes) [3]. Although flux rates and intermediate metabolite concentrations are generally regulated by enzyme and transporter activities, some physiological processes involving metabolic fluxes can be driven by metabolites and nutrient concentrations. An example is the regulation of mitochondrial respiratory flux in beta cells by extracellular levels of glucose (reviewed in Liesa and Shirihai, 2013). Thus, in this study, we aimed to test whether changes in the extracellular redox state are able to regulate normal tissue metabolism. As most of these physiological metabolites that constitute the circulating redox state can have other effects that are independent of communicating changes to the intracellular NADH/NAD^+^ redox state, we focused our studies on liver and the ratio of the ketone bodies βOHB/Acoc, as liver can produce ketone bodies but it cannot consume or use them as fuels. This known characteristic of hepatocytes predicts that the effects on liver of the extracellular changes in the βOHB/Acoc ratio will not be a result of their intracellular catabolism. Thus, we aimed to assess how changes in the extracellular βOHB/Acoc ratio and other redox pairs affect the internal redox state, mitochondrial respiratory function, ROS production, and liver function in freshly isolated murine hepatocytes. In this regard, we found that the extracellular redox state can affect gluconeogenesis and glycogen synthesis while regulating mitochondrial function and ROS activity. Thus, we demonstrate for the first time that the extracellular redox state constituted by physiological metabolites can regulate intracellular metabolism in liver.

## Materials and Methods

### Materials

Chemicals and reagents were purchased from Sigma-Aldrich (St. Louis, MO) unless otherwise noted. A 50/50 mixture of d- and l-β-hydroxybutyrate was used; however, since only the d enantiomer is convertible to Acoc by βOHB dehydrogenase, the concentration of βOHB was doubled to yield an appropriate concentration of d-βOHB. The only commercially available form of acetoacetate available was lithium acetoacetate, so a lithium chloride control was performed where appropriate to correct for the potentially confounding effects of lithium. Lithium chloride at the relevant concentrations tested did not affect any of the dependent variables assessed in this paper (*p* > 0.05, data not shown). The concentration of ketone bodies in human plasma can be up to 7 mM and under ketosis, it can reach 25 mM. In our system, we used a total concentration of ketone bodies of 20 mM, which is in the high-end concentration that mouse hepatocytes can potentially be exposed to (2.8 fold to fasting concentrations). The concentration of cysteine in normal individual plasma can be 250 μM and we used 200 μM, therefore cysteine was under the same range. The concentration of glutathione in mouse plasma can be around 20 μM for GSH and 3–5 μM for GSSG (Leeuwenburgh C and Ji LL 1998) and we used 100 μM + 10 μM (GSH/GSSG). Clinical supplies and cell culture supplies (e.g. syringes, plates) were purchased from Thermo Fisher Scientific (Waltham, MA), Becton Dickenson (Hunt Valley, MD), or Invitrogen Life Technologies (Carlsbad, CA) unless otherwise noted.

### Ethics Statement

All animal experiments were overseen and approved by the Institutional Care and Use Committee at Boston University (Protocol no. AN-14071). The protocols were in strict accordance with the Guide for the Care and Use of Laboratory Animals and care was taken to minimize pain and suffering of the animals [10].

### Hepatocyte isolation and culture

Male C57BL/6NCrl mice were purchased from Charles River Laboratories (Wilmington, MA) at 6–8 wks old. These mice originate from the NIH strain of the C57BL/6 mouse. Mice were euthanized with carbon dioxide, then hepatocytes were freshly isolated using the following protocol. The liver was cannulated and perfused through the inferior vena cava with Krebs buffer solution (118 mM sodium chloride, 4.8 mM potassium chloride, 25 mM sodium bicarbonate, 1.2 mM potassium phosphate, 1.2 mM magnesium sulfate, pH 7.2) supplemented with 0.5 mM ethylene glycol tetraacetic acid and 5 mM glucose for approximately 5 minutes using a peristaltic pump at a rate of 4 mL per minute. As soon as proper perfusion of the liver was confirmed with a tissue color change, the portal vein was cut to allow the complete clearing of blood from the liver and the cannula was secured with a suture around the vena cava. To prepare liver digestion media, type 4 collagenase (*Clostridium histolyticum*; Worthington Biochemical Corp., Lakewood, NJ) was first dissolved in 10 mL of 60 mM calcium chloride at 100 U/mL. Then, the collagenase solution was diluted into 35 mL of Krebs solution with 2.5 mM calcium chloride. The liver was then perfused with digestion media until hepatocytes were released from the connective tissue. The cell suspension was subsequently filtered through a mesh cell strainer (70 μm nylon) and centrifuged at 39.1 *g* for 2 min. The supernatant was discarded and the cell pellet was resuspended in 10 mL Percoll and 10 mL of Krebs with 2.5 mM calcium chloride. The cells were centrifuged again and the pellet was resuspended in 5 mL of plating media (Dulbecco's modified Eagle's medium (DMEM), 2 mM pyruvate, 10% fetal bovine serum (FBS), 100 nM insulin, 10 mM glucose, 100 nM dexamethasone, and 1% penicillin/streptomycin). Hepatocyte viability was determined with a hemocytometer and trypan blue exclusion. Only hepatocyte isolations of over 80% viability were used. Hepatocytes were seeded onto collagen-coated plates at a density of 12 × 10^4^ cells per well in a 48-well plate (1 cm^2^ growth area) and 25 × 10^4^ cells per well for a 24-well plate (2 cm^2^ growth area) for high cell confluency. The plates were incubated at 5% carbon dioxide and 37°C. After 2 h, plating media was replaced to decrease the number of unattached, nonviable hepatocytes. After an additional 4 h, the cells were firmly attached to the plates in a monolayer.

### Determination of the internal redox state and ROS production

For internal redox state determination, cells were suspended in Krebs-HEPES buffer (20 mM HEPES, 119 mM sodium chloride, 4.6 mM potassium chloride, 1 mM magnesium sulfate, 0.15 mM sodium phosphate, 0.4 mM potassium phosphate, 5 mM sodium carbonate) right after the isolation. Cells were placed in a fluorimeter cuvette at a density of 1 x 10^6^ cells/mL and stirred at 37°C while fluorescence was monitored. After a baseline was established, the compounds of interest were injected into the cuvette. NAD(P)H autofluorescence units were measured with a fluorescence spectrophotometer (F-2000, Hitachi, Tokyo, Japan), which mainly reflect mitochondrial NAD(P)H [11].

ROS measurements were performed with the hydrogen peroxide-sensitive dye 2',7'-dichlorodihydrofluorescein diacetate (H_2_-DCF-DA; Invitrogen Molecular Probes, Eugene, OR) at a working concentration of 2 μM. Freshly isolated hepatocytes were cultured in 24-well plates maintained at 37°C throughout the experiment. Five hours after isolation, cells were loaded with H_2_-DCF-DA dye for 30 min. Cells were washed twice for 10 min with Krebs-HEPES buffer and, subsequently, treated with the redox couples at the indicated ratios in Krebs-HEPES buffer. DCF fluorescence was measured at 40 min using a TECAN M1000 plate reader (Research Triangle Park, NC).

### Measurement of lipid peroxidation

Freshly isolated hepatocytes were incubated overnight in DMEM media with 10 mM glucose in the absence of FBS. The next day, the cells were treated for 2 h with the compounds of interest in Krebs-HEPES buffer. Lipid peroxidation was then assessed using a modified TBARs assay as follows. After incubation in the experimental compounds, cells were exposed to a solution containing 0.4% 2-thiobarbituric acid and 10% acetic acid. Sodium hydroxide was added to achieve a final concentration of 0.0625 N. Standard curves were produced by serial diluting 1,1,3,3-tetraethoxypropane. The cell solution was incubated at 90°C for 60 min, then cooled and centrifuged at 15,000 *g* for 5 min. The supernatant was isolated and fluorescence was measured on the TECAN M1000 plate reader (Männedorf, Switzerland) with an emission wavelength of 553 nm and an excitation wavelength of 515 nm.

### Measurement of mitochondrial respiration

Hepatocytes were plated in collagen-coated Seahorse V.7 multi-well culture plates. Exactly 2 h later, plating media was replaced by running media (XF Seahorse Assay Media supplemented with 3 mM glucose and 20 mM HEPES) and the plate was placed at 37°C for 1 h (no carbon dioxide). Oxygen consumption was measured at 37°C using a Seahorse XF24 analyzer with a companion extracellular oxygen flux sensor (Seahorse Bioscience, Billerica, MA). Redox couples were injected through port A, whereas mitochondrial stress test compounds (2 μM oligomycin, 0.25 μM FCCP, and 1 μM antimycin A) were injected through ports B, C, and D, respectively, to measure mitochondrial respiration linked to ATP synthesis, leak, maximal respiratory capacity and non-mitochondrial oxygen consumption.

### Endogenous glucose production and glycogen synthesis

Cells were cultured overnight in DMEM without glucose or FBS. For endogenous glucose production measurements, cells were pre-treated the following day with the compounds of interest in Krebs-HEPES buffer for 45 min after which 10 mM l-alanine was added as gluconeogenic substrate for 3.5 h. Glucose was measured from the media using the endpoint fluorimeter coupled enzyme assay previously described [12]. For glycogen synthesis measurements, except for the controls, cells were treated with 30 mM glucose in the presence of the compounds of interest for 3 h. Then, cells were harvested in 30% potassium hydroxide and boiled for 15 min. Glycogen was precipitated with 66% ethanol overnight at -20°C and later converted to glucose with α-amyloglucosidase. Glucose was then measured using the same protocol.

### β-oxidation measurement

Cells were plated in 24-well plates and incubated overnight in DMEM without glucose or FBS. The following day, cells were pre-incubated with the compounds of interest in Krebs-HEPES buffer for 1 h before the initiation of the assay. ^14^C-Oleate oxidation assays for measuring fatty acid oxidation activity were performed as previously described [13,14]. Briefly, cells were incubated with the compounds of interest in the presence of 500 μL/well of modified Krebs-HEPES buffer containing 3 mM glucose and 12.5 μM ^14^C-oleate (54 mCi/mmole, Perkin Elmer). A piece of 1.5 cm-diameter Whatman filter paper was suspended above each well and the plate was sealed for 2 h. After the incubation period, the filter paper was wetted with β-phenethylamine followed by acidification of the media above the cells with 100 μL/well of 6 M sulfuric acid. The cell plate remained sealed for an additional hour in order to trap the ^14^C-labeled carbon dioxide produced during the incubation period onto the filters. Filter paper was collected and suspended in scintillation fluid (Ecoscint, National Diagnostics) and β particle emission was analyzed using a LabLogic 300SL Liquid Scintillation Counter (Brandon, FL).

### Statistics

All data were pooled from at least 3 replicates of at least 3 independent experiments. The mean ± standard error of the mean is reported, unless otherwise noted. Comparison of means was accomplished with analysis of variance (ANOVA) with a Tukey’s honestly significant difference (HSD) posthoc analysis, where appropriate, and statistical significance was determined at *p* < 0.05 (JMP10, 2012, SAS Institute Inc., Middleton MA).

## Results

### Extracellular ketone bodies can modulate the NAD(P)H redox state

**Fig 1:**
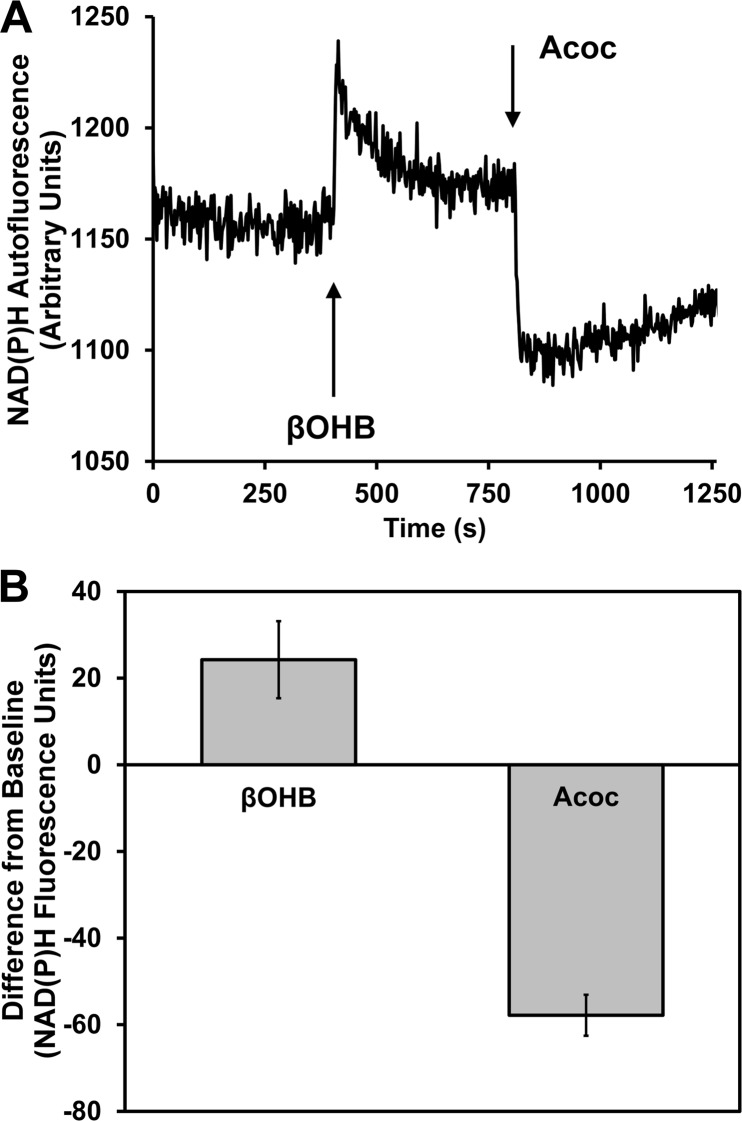
External addition of reduced or oxidized ketone bodies affect the internal redox state. NAD(P)H fluorescence was measured within hepatocytes in suspension using a fluorometer. A) Changes in fluorescence correspond to the addition of external, sequential addition of 40 mM of a 50/50 mixture of l- + d-βOHB or 20 mM Acoc at the points indicated. This trace is one representative trace of three independent experiments. B) Quantification of the change in arbitrary fluorescence units upon addition of βOHB or Acoc to baseline (without previous addition of either ketone body; *n* = 5 independent experiments, *p* = 0.003, *t*-test).

Hepatocytes produce ketone bodies from fatty acid β-oxidation in the mitochondria during starvation, which are exported to the bloodstream through the monocarboxylate transporter (MCT1) and then used as fuels by other tissues. Hepatocytes do not consume ketone bodies and therefore, they have no physiological need to import them. In order to assess whether extracellular βOHB and Acoc may affect the intracellular NAD(P)^+^/NAD(P)H ratio in intact hepatocytes, NAD(P)H fluorescence was measured after adding βOHB or Acoc in primary mouse hepatocytes in suspension. Fig. 1A illustrates a representative trace of NAD(P)H fluorescence and the changes upon addition of βOHB and Acoc; Fig. 1B shows the mean change in NAD(P)H fluorescent units from baseline compiled from five independent experiments. Upon addition of βOHB, NAD(P)H fluorescence increased likely due to migration of the equilibrium from βOHB and NAD^+^ towards Acoc and NADH in the mitochondria via βOHB dehydrogenase [15]. This trace is similar to what was observed by Latipää *et al*. in isolated mitochondria from liver [16] and by our group in permeabilized β-cells [11]. This initial burst, which then decreased but still maintained a more reduced redox state than baseline (Fig. 1A, B), indicated that changes in the extracellular redox state were rapidly communicated inside the cell (initial burst) and that the decay was a result of adaptation of the cell and the mitochondria to the new extracellular redox state, resulting in a new equilibrium, with a more reduced state and increased respiratory activity. Acoc rapidly decreased NAD(P)H fluorescence, presumably due to conversion of Acoc and NADH to βOHB and NAD^+^. Therefore, these data demonstrate that extracellular ketone bodies can modulate the NAD(P)H redox state in a similar manner to what was shown in isolated mitochondria and permeabilized β-cells. It also suggests that ketone bodies might enter the hepatocytes despite their lack of need for them as a fuel.

### Extracellular redox pairs modulate ROS production

**Fig 2:**
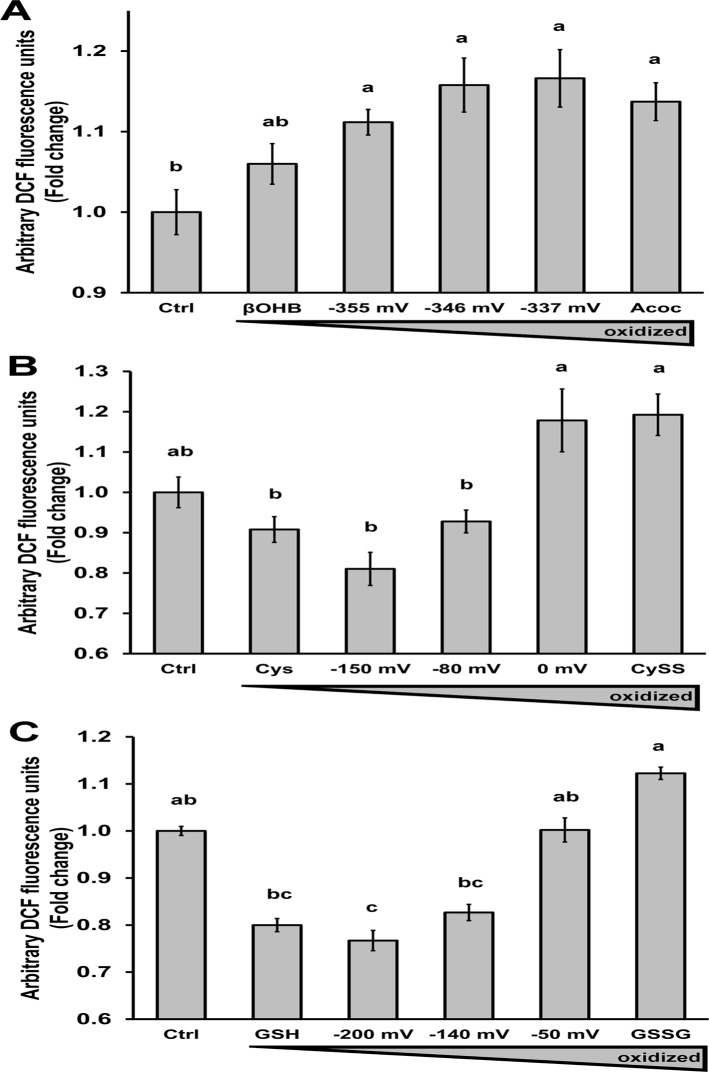
Extracellular incubation with increasingly oxidized ratios of redox couples increased ROS production. Intracellular ROS measurements in primary cultured hepatocytes treated with A) 20 mM total active ketone bodies (-355 mV = 2:1 d-βOHB:Acoc; -346 mV = 1:1 d-βOHB:Acoc; -337 mV = 1:2 d-βOHB:Acoc), B) 200 μM cysteine (cysteine + cystine), or C) 110 μM glutathione (reduced + oxidized glutathione). Cells were plated in 24 well plates. Five hours later, the cells were loaded with H_2_-DCF-DA for 30 min, rinsed, and then treated with the compounds of interest. Data represent the fluorescence from DCF after 40 min of treatment with the compounds. Data represent avg ± SE of three independent experiments. Different letters indicate statistical significance, ANOVA, Tukey’s posthoc analysis.

Given the intimate connection between changes in the intracellular redox state and ROS, we next investigated whether the external redox state can directly affect internal ROS production in primary hepatocytes. DCF is a ROS-sensitive dye that fluoresces according to hydrogen peroxide (H_2_O_2_) levels. In addition to βOHB/Acoc, we tested cysteine/cystine, and GSH/GSSG and their ratios are expressed as the steady-state redox potential calculated from the Nernst equation with the standard cell potential (*E*_*0*_) of -346 mV for βOHB/Acoc, -264 mV for GSH/GSSG, and -250 mV for cysteine/cystine. Addition of redox pairs with increasing oxidative potential led to increases in DCF fluorescence for ketone bodies (Fig. 2A), cysteine/cystine (Fig. 2B), and reduced/oxidized glutathione (Fig. 2C). These data demonstrate that the external redox potential conveyed by ketone bodies or thiol redox couples cyst(e)ine and glutathione can all impact ROS production.

To get a better understanding of these changes in ROS levels induced by the extracellular redox state, we studied the effects of the different redox ratios on oxidative damage. To this end, we measured lipid peroxidation, as a transient increase in ROS can lead to an auto-amplifiable lipid peroxidation of unsaturated lipids. Addition of βOHB and Acoc in various ratios did not increase lipid peroxidation (S1 Fig.). Furthermore, when *tert*-butyl hydroperoxide (tBH), an inducer of hydrogen peroxide production, was added, βOHB attenuated lipid peroxidation (*p* < 0.05), but not to as large an extent as *N*-acetylcysteine (NAC), a precursor to glutathione (S1 Fig.). This would be consistent with the increase in NAD(P)H caused by βOHB, which could promote degradation of H_2_O_2_ by increasing the activity of NADPH-dependent peroxidases.

### A more reduced pool of ketone bodies increases basal mitochondrial respiration and maximal respiratory capacity

**Fig 3:**
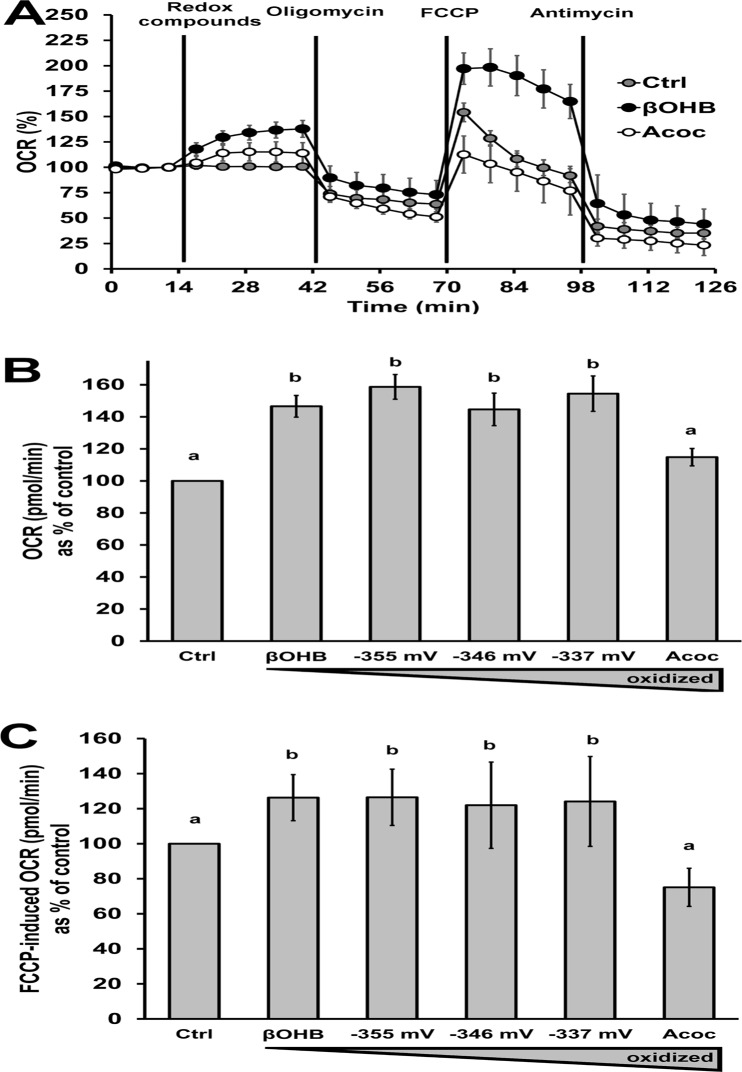
Addition of a more reduced pool of ketone bodies increases basal mitochondrial respiration and maximal respiratory capacity. A: Example trace of the oxygen consumption rate (OCR) as a percentage of the baseline as a function of time. Redox compounds at 0 mM (control, gray) or 20 mM ketone bodies (d-βOHB, black, or Acoc, white), 2 μM oligomycin, 0.25 μM FCCP, and 1 μM antimycin A were sequentially added at the time points indicated. Each point shows the avg ± SE of 3 or 4 replicates. This trace is one representative trace of 4 independent experiments. B: Quantification of the increase in OCR upon addition of the redox compounds normalized to the control. C: Maximal respiratory capacity relative to the control. For B and C, raw OCR values after addition of antimycin A were subtracted before further analysis, as they correspond to non-mitochondrial respiration. B and C data represent avg ± SEM of 4 independent experiments. Different letters indicate statistical significance (*p* < 0.05, ANOVA, Tukey’s posthoc analysis).

Because external βOHB and Acoc can affect NAD(P)H levels and thus the mitochondrial redox state (Fig. 1), we next directly investigated mitochondrial respiration upon exposure to these compounds. In this case, the main nutrients present in the media were glucose and amino acids, without exogenous free fatty acids. Acute addition of βOHB with or without Acoc increased basal respiration, whereas Acoc alone only caused a slight increase in this parameter (Figs. 3A and 3B). This result is interesting because ratios of βOHB/Acoc had opposite effects on NAD(P)H redox state (Fig. 1) and ketone bodies are substrates for respiration in many tissues including the heart [17], though hepatocytes do not use ketone bodies as fuel. In regard to maximal respiratory capacity, βOHB led to a 26% increase, while Acoc led to a 25% decrease compared to the control (Fig. 3C). Similar to the stimulation over basal respiration, maximal respiration capacity was similar between pure βOHB and all ratios of βOHB/Acoc in which βOHB was present (*p* > 0.05). The respiratory decline over time after FCCP injection in control and Acoc-treated cells reflected the expected decrease in fuel availability with increased respiratory rates and thus, the absence of sufficient NADH to maintain maximal respiration. On the other hand, under βOHB, this decline is prevented because βOHB increases NADH availability in the mitochondrial matrix. When assessing the mitochondrial proton leak relative to the control, there was no significant effect of βOHB/Acoc ratio (*p* > 0.05, data not shown). Similarly, respiration linked to ATP synthesis was not altered by the identity of the ketone body when considering and when ignoring the effect of the redox compounds on basal respiration (*p* > 0.05, data not shown). This result demonstrates that addition of ketone bodies increases the ATP demand in hepatocytes, as the increase that they induced is fully inhibited by oligomycin. Furthermore, the fact that both βOHB and Acoc increased basal mitochondrial respiration despite opposite effects on the redox state suggests that the extracellular redox state could be changing the fuel oxidized for mitochondrial ATP synthesis.

### The external redox state affects β-oxidation

**Fig 4:**
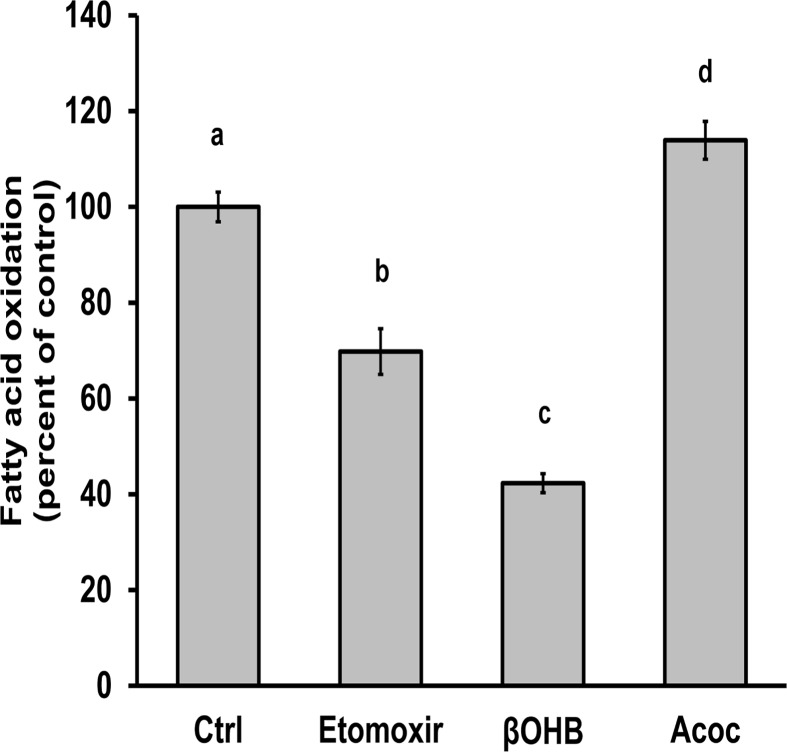
βOHB inhibits β-oxidation and Acoc accentuates β-oxidation. Freshly isolated hepatocytes were pre-incubated with 10 mM of a 50/50 mixture of l- and d-βOHB, 5 mM Acoc, or neither for 1 hour. ^14^C-labeled oleate was subsequently added for a 2 hour incubation in the presence of the treatments, 30 μM etomoxir, or neither (control, Ctrl). ^14^C from ^14^C-labeled carbon dioxide was detected and reported as a percentage of the control. Differences between all treatments are statistically significant (*p* < 0.05, ANOVA, Tukey’s posthoc analysis).

In order to assess the effects of the external redox state on the fuel used for respiration, freshly isolated primary hepatocytes were subjected to βOHB or Acoc and conversion of exogenous oleate into carbon dioxide was determined. Etomoxir is an inhibitor of carnitine palmitoyltransferase 1 (CPT1), which catalyzes the rate-limiting step in the mitochondrial import of fatty acids, and thereby a strong inhibitor of fatty acid oxidation [18]; it was used here as a positive control. Illustrated in Fig. 4, etomoxir inhibited fatty acid oxidation 30% (*p* < 0.05), and βOHB inhibited fatty acid oxidation even further, approximately 60% (*p* < 0.05). On the other hand, Acoc stimulated fatty acid oxidation 14% (*p* < 0.05). These data show that the external redox state can greatly affect the regulation of fatty acid β-oxidation, presumably via the mitochondrial redox change [11].

### A more reduced redox state stimulated endogenous glucose production and glycogen synthesis

**Fig 5:**
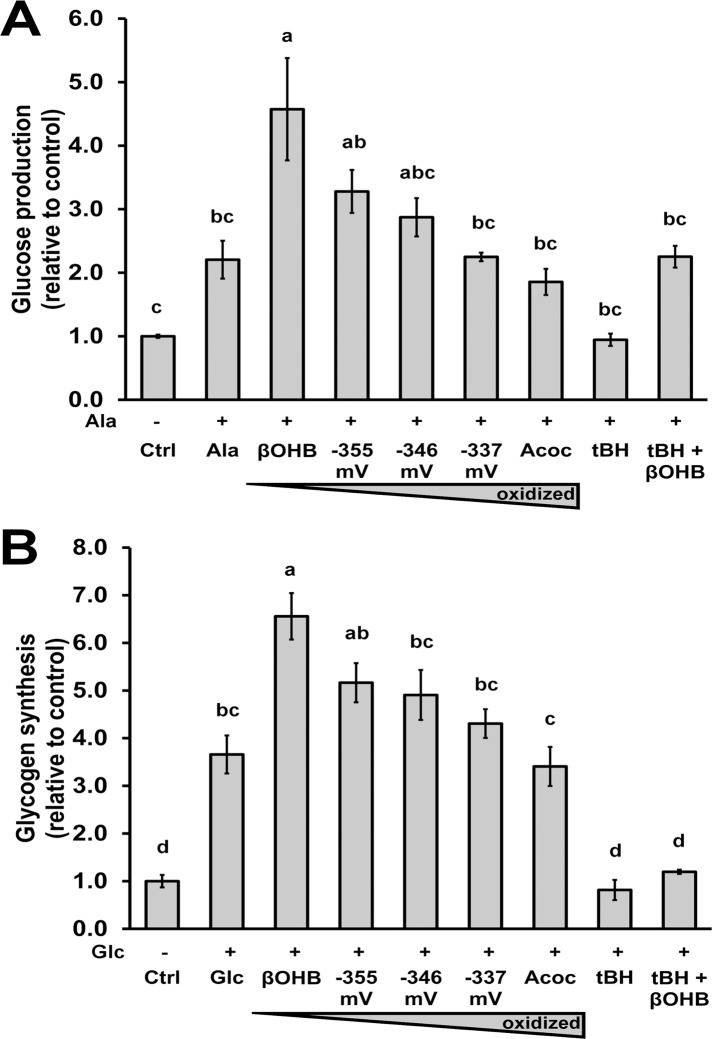
A more reduced redox state stimulates endogenous glucose production and glycogen synthesis. Cells were incubated in Krebs buffer only (control, Ctrl), with 10 mM l-alanine (Ala) as a gluconeogenic substrate for 3.5 hours to assess glucose production (A), or with 30 mM glucose (Glc) as a glycogen substrate to assess glycogenesis (B). All treatments included alanine (A) or glucose (B) except for the control in addition to: 20 mM total active ketone bodies (-355 mV = 2:1 d-βOHB:Acoc; -346 mV = 1:1 d-βOHB:Acoc; -337 mV = 1:2 d-βOHB:Acoc), and/or 40 μM *tert*-butyl hydroperoxide (tBH). Glucose was measured from the media (A) and glycogen was measured from hepatic stores (B). Data represent Avg ± SE of 3–6 independent experiments. Different letters signify statistical significance (*p* < 0.05, ANOVA, Tukey’s posthoc analysis).

Two of the major functions of the liver are endogenous glucose production (gluconeogenesis) and glycogen synthesis. Gluconeogenesis occurs during starvation, whereas glycogen synthesis occurs during fed states and can be stimulated in fasted hepatocytes by the addition of glucose. However, these hormonally antonymic processes are enhanced by the same factors, such as increased ATP synthesis and mitochondrial NADH availability. In order to study whether or not the external redox state can also affect these functions, we assessed these processes in hepatocytes under a range of βOHB/Acoc ratios. Additions of ketone bodies in ratios that achieved a more reduced state were sufficient to increase glucose production (Fig. 5A) and glycogen synthesis (Fig. 5B). tBH was used to promote oxidative stress and ROS production in the hepatocytes. tBH effectively reduced both glucose production and glycogen synthesis 60% and 80% compared to conditions where only alanine or glucose were used (*p* < 0.05; Fig. 5). Addition of βOHB to the hepatocytes stressed with tBH restored glucose production to the same levels as alanine (Fig. 5A) but was not successful restoring glycogen synthesis, despite a non-statistically significant increase over tBH alone (Fig. 5B). Together, these data suggest that the βOHB/Acoc ratios are affecting ROS (Fig. 2) involved in signaling (e.g. gluconeogenesis and glycogen synthesis, Fig. 5), but not ROS that damages the cell, as demonstrated by the lack of changes in lipid peroxidation (S1 Fig.). The effects of GSH/GSSG and cysteine/cystine on endogenous glucose production and glycogen synthesis were also assessed but no changes were observed among the conditions (data not shown).

## Discussion

Herein, we showed that alterations in the extracellular redox state, which are known to occur in diabetes [9], aging [19], cardiac dysfunction [20], and other disorders, can directly alter the internal redox state and the function of healthy murine hepatocytes. Changes in the extracellular ratio of βOHB/Acoc were indeed sufficient to regulate intracellular ROS, mitochondrial respiration, fatty acid oxidation, glucose production, and glycogen synthesis in hepatocytes.

We propose that the mechanism by which a greater ratio of βOHB/Acoc allows for protection of liver function from oxidative damage caused by tBH (Fig. 2A), changes in fatty acid oxidation (Fig. 4), and a greater mitochondrial respiration capacity (Fig. 3) may occur via the mitochondrial transhydrogenase (NNT). Different mitochondrial redox reactions, but specifically the electron transfer through complexes I and III, are a significant generator of superoxide radicals from molecular oxygen. These radicals are scavenged by superoxide dismutase (SOD) into hydrogen peroxide that glutathione peroxidases can reduce to water using GSH as an electron donor. Mitochondrial glutathione reductase requires NADPH as an electron donor to regenerate GSH from GSSG. It is estimated that at least 45% of the NADPH in the mitochondria is generated from NADH by the transhydrogenase [21]. We conjecture that βOHB supplies NADH and, consequently NADPH via the transhydrogenase, affording the cell more robust redox regulation.

It was shown directly that external βOHB increased and Acoc decreased the amount of reduced NAD(P)H in hepatocytes (Fig. 1). This is consistent with other studies performed in other cell types that have shown that alteration of extracellular βOHB or Acoc in permeabilized β-cells [11] and isolated mitochondria [16]; extracellular cysteine/cystine ratio (reviews: [1,22]); or extracellular Lac/Pyr ratio [23,24] can alter intracellular redox-sensitive proteins, intracellular signaling, and/or cellular function. In addition, a more reduced ratio of ketone bodies, cyst(e)ine, and glutathione led to less intracellular ROS production than more oxidized ratios of the same pairs (Fig. 2), likely due to enhanced ratio of reduced/oxidized glutathione. Interestingly, the addition of different ratios of ketone bodies maintained a better proportionality to DCF fluorescence than GSH/GSSG and cysteine/cystine pairs. Ketone bodies cannot be consumed in hepatocytes and would exclusively affect mitochondrial redox state, whereas GSH and cysteine can be consumed by hepatocytes and can affect the redox state of multiple subcellular compartments. The inherently different properties of these redox pairs could explain why some proportionality is lost when GSH/GSSG and cysteine/cystine are brought to the non-physiological extremes. Moreover, the capacity of the redox pairs to alter ROS levels can also be modulated by other antioxidant systems not directly dependent on the NAD(P)H redox state (i.e. catalase, SOD, etc.), thus there might be a point at which further decreases in NAD(P)H, through changes in the extracellular redox state, might not increase ROS, suggesting a threshold effect. Consistent with our data, shifting of the extracellular redox state to a more oxidized state by decreasing the cysteine/cystine ratio led to increased ROS production in other models [25], [26] [27]. Despite the observed effects of GSSG/GSH and Cys/Cyss ratios on ROS production (Fig. 2B, 2C), we did not detect a consistent effect on gluconeogenesis and glycogen synthesis by these redox pairs (data not shown), as we did see with βOHB/Acoc. Our main explanation for this difference between the extracellular redox pairs is that ketone bodies are exclusively mitochondrial extracellular redox pairs, as the enzyme that interconverts them is mitochondrial and they cannot be metabolized by hepatocytes. Thus the βOHB/Acoc effect is mostly dependent on their effects on the mitochondrial NAD(P)H redox state. On the other hand, cysteine can be consumed for protein synthesis or amino acid metabolism, and reduced or oxidized glutathione (the latter through glutaredoxin activity) can be used to glutathionylate proteins. Furthermore, GSSG/GSH and cysteine/cystine mediated changes can potentially occur in all cellular compartments, not only in mitochondria. Therefore, these additional effects of GSSG/GSH and cysteine/cystine might be masking the expected effects on glycogen synthesis and gluconeogenesis through their potential effect modulating the intracellular and mitochondrial redox state.

Mitochondrial function is critical for metabolic health and dysfunction of mitochondria is implicated in obesity, diabetes, and other metabolic disorders [28]. Under certain circumstances, mitochondria can be major sites of ROS production. As both generators of ROS and responders to ROS signals, mitochondria require a delicate balance between ROS generation and ROS quenching [29]. Whereas Maalouf *et al*. observed that addition of ketone bodies in a ratio of 1:1 βOHB:Acoc increased mitochondrial respiration compared to no addition of ketone bodies in neurons [30], we extended this findings to hepatocytes which, unlike neurons, do not use ketone bodies as fuels. Indeed, we observed that βOHB and the more reduced ratios of βOHB/Acoc enhanced basal respiration (Fig. 3B) and maximal capacity of mitochondrial respiration (Fig. 3C) in intact hepatocytes. Because mitochondrial respiration is a direct source of ROS, introduction of more reduced compounds (e.g. βOHB) may permit both more respiration and protection against excess mitochondrial ROS generation via higher availability of mitochondrial NADPH for glutathione peroxidases through the activity of the mitochondrial transhydrogenase (NNT).

Fatty acid oxidation is regulated in part by the redox state of the mitochondria. One cycle of fatty acid oxidation generates two reducing equivalents- one in the form of NADH and one in the form of an electron-transferring flavoprotein (review: [31]). The third step in fatty acid oxidation, specifically, is performed by a set of 3-hydroxyacyl-CoA dehydrogenases (HADH) which are oxidoreductases that convert 3-hydroxyacyl-CoA and NAD^+^ to 3-ketoacyl-CoA and NADH. The mitochondrial NAD^+^/NADH ratio can serve as feedback control for this enzyme, and Acoc-generated NAD^+^ could increase activity of HADH while βOHB-generated NADH could allow inhibition [31].

Key metabolic functions of the liver are to provide fuel to other tissues and to store energy; specifically to produce glucose and secrete ketone bodies in the fasting state, and synthesize glycogen in the fed state. Alteration of the extracellular redox state via manipulation of cysteine/cystine ratio has been well established to affect function in other cell types [1]; for example, a more oxidized cysteine/cystine ratio induces pro-inflammatory signaling by endothelial cells, thereby contributing to cardiovascular disease (review: [8]). This study extends those findings to ketone bodies and hepatocyte functions, illustrating that a more reduced extracellular state led to elevations in alanine-stimulated glucose production and glucose-stimulated glycogen synthesis (Fig. 5). We propose that the extracellular redox state can positively modulate these two hormonally opposed metabolic pathways, as they have common positive effectors. Rates of gluconeogenesis and glycogenesis both depend chiefly on the precursor pool and energy state of the hepatocyte and are directly driven by the mitochondrial and cytosolic NAD^+^/NADH ratio. An increase in mitochondrial NADH together with an increase in mitochondrial respiration, will lead to an increase in ATP synthesis, which is used for the conversion of glucose into glucose-6-phosphate, the first step of glycogen synthesis, and also to elongate glycogen. Gluconeogenesis is regulated by the nicotinamide redox state at the level of glyceraldehyde-3-phosphate dehydrogenase (GAPDH), which converts 1,3-bisphosphoglycerate and NADH to glyceraldehyde-3-phosphate and NAD^+^, and also by the reversible malate-aspartate shuttle ([32,33] and references within). Specifically, mitochondrial NADH is required to efficiently transform mitochondrial pyruvate to cytosolic oxaloacetate, required for gluconeogenesis. Mitochondrial generation of NADH by βOHB and NAD^+^, via βOHB dehydrogenase in the mitochondrial matrix, is consistent with the observed stimulated flux through the gluconeogenic pathway (Fig. 5). Indeed these different requirements for NADH and ATP by gluconeogenesis and glycogen synthesis respectively are also illustrated by the tert-butyl-hydrogen peroxide treatments (tBH). One of the main effects of low tBH concentration treatment (40 μM), in addition to causing oxidative damage, is to oxidize NADPH for ROS detoxification. This oxidation of NADPH limits the availability of NADH for gluconeogenesis and thus should slow down its rate. However, by providing βOHB under the presence of tBH, NAD(P)H availability is increased and thus it limits the competition for NADPH use between gluconeogenesis and ROS detoxification. On the other hand, in the presence of tBH, the increased NADH availability by βOHB is not sufficient to produce ATP at the faster rates required for glycogen synthesis (characteristic of starved hepatocytes treated with 30 mM glucose), as shown in Fig. 5. Why is mitochondrial ATP synthesis more limited than NADH availability under tBH treatment? We think that this is a consequence of the uncoupling activity of the mitochondrial transhydrogenase (NNT). The transhydrogenase interconverts NADH to NADPH in the mitochondria. This NNT-mediated NADPH production translocates protons across the inner membrane, which can no longer be used for mitochondrial ATP synthesis. We expect that interconversion of NADH to NADPH should increase with tBH treatment to detoxify ROS through NADPH dependent peroxidases. By adding βOHB, NADH availability for gluconeogenesis is increased in the mitochondria, but this also predicts that the transhydrogenase will also work faster producing NADPH, therefore limiting mitochondrial ATP synthesis at higher rates. As glycogen synthesis is more sensitive to ATP availability than gluconeogenesis, we think this is the reason why βOH treatment can partially rescue gluconeogenesis but not glycogen synthesis in the presence of tBH (Fig. 5A and B).

In conclusion, we show that increasing the NAD(P)H/NAD(P)^+^ using extracellular ketone bodies in the presence of alanine, is sufficient to enhance gluconeogenesis. Therefore, these data provide evidence for a potentially novel mitochondrial mechanism to regulate hepatic glucose production.

Understanding the regulation and communication of the intracellular and extracellular redox state will allow us to make more accurate diagnoses (e.g. diabetes, as suggested by Ido *et al*. [34]), predict/monitor the success of treatments [35,36], identify lifestyle habits that benefit or abuse the redox state (e.g. exercise and cigarette smoking, respectively), develop targeted therapeutics to ameliorate redox insults, and develop pharmaceuticals to aid in the maintenance of redox regulation and/or communication. In fact, therapeutic potential for redox manipulation has been recognized for treatment of many chronic diseases including diabetes [37], cardiovascular disease [38], cancer [39], and lung disease [40]. However, despite promising *in vitro* results, the success of redox treatments has so far been disappointing (reviews: [41,42]). Limited success of treatments may be because of the approach to indiscriminately quench ROS and not to rectify the cause(s) to the shift in redox balance, using an antioxidant that is too strong or not strong enough, or other drawbacks. Because ROS signaling is essential for various processes such as insulin secretion [43,44], broadly quenching ROS may not ameliorate the ailment, and may have negative consequences [41,42].

The success of redox-based treatments hinges on the ability to better understand the compartmentalization and crosstalk between redox-regulation machinery and redox-sensitive pathways. Herein, we evaluated the effects of the external redox environment on various redox regulation mechanisms, ROS generation, and hepatocyte function. We showed that external ratios of ketone bodies (βOHB/Acoc) can communicate with the cytosolic and mitochondrial compartments of hepatocytes, thereby affecting ROS generation, cellular metabolism, and hepatocyte function.

## Supporting Information

S1 Fig The extracellular redox state did not affect lipid peroxidation. Cells were incubated overnight in the presence of 10 mM glucose and in the absence of FBS. Treatments were then introduced in Krebs buffer for 2 hours and lipid peroxidation was measured as described in the materials and methods. The control (Ctrl) condition was Krebs buffer only. Ketone bodies were introduced to yield a total active concentration of 20 mM (d-βOHB + Acoc; -355 mV = 2:1 d-βOHB:Acoc; -346 mV = 1:1 d-βOHB:Acoc; -337 mV = 1:2 d-βOHB:Acoc). *N*-acetylcysteine was added at 10 mM and *tert*-butyl hydroperoxide (tBH) was added at 40 μM where indicated. Data represent Avg ± SE of 3–6 independent experiments. Different letters indicate statistical significance (*p* > 0.05, ANOVA, Tukey’s posthoc analysis).
(TIFF)
