# Supplementary figures and images for: The Extracellular Redox State Modulates Mitochondrial Function, Gluconeogenesis, and Glycogen Synthesis in Murine Hepatocytes
Source: PLoS One. 2015 Mar 27;10(3):e0122818. doi: 10.1371/journal.pone.0122818 (PMC4376787; doi:10.1371/journal.pone.0122818)

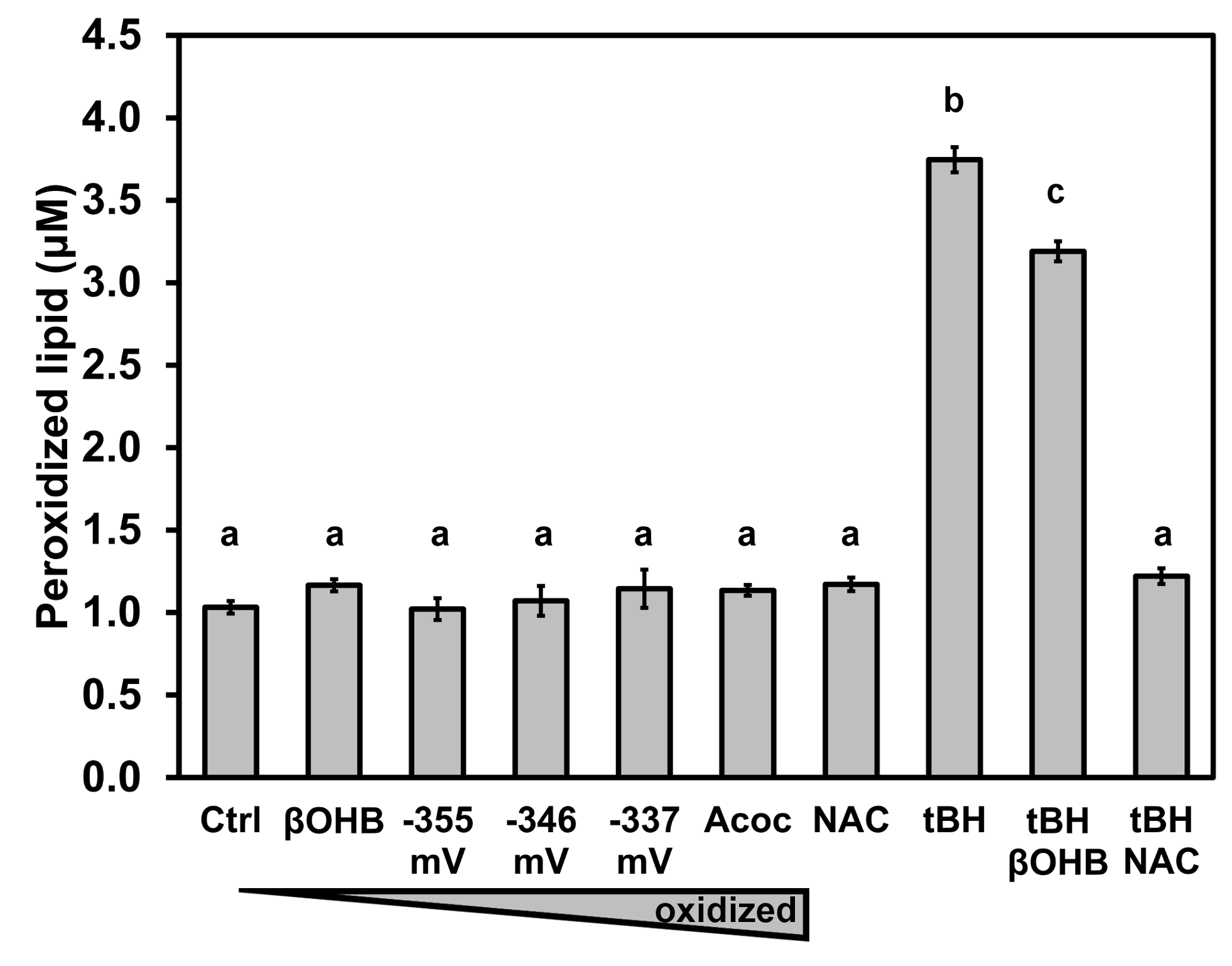

Supplement: S1 Fig — Cells were incubated overnight in the presence of 10 mM glucose and in the absence of FBS. Treatments were then introduced in Krebs buffer for 2 hours and lipid peroxidation was measured as described in the materials and methods. The control (Ctrl) condition was Krebs buffer only. Ketone bodies were introduced to yield a total active concentration of 20 mM (d-βOHB + Acoc; -355 mV = 2:1 d-βOHB:Acoc; -346 mV = 1:1 d-βOHB:Acoc; -337 mV = 1:2 d-βOHB:Acoc). N-acetylcysteine was added at 10 mM and tert-butyl hydroperoxide (tBH) was added at 40 μM where indicated. Data represent Avg ± SE of 3–6 independent experiments. Different letters indicate statistical significance (p > 0.05, ANOVA, Tukey’s posthoc analysis). (TIFF) [file pone.0122818.s001.tiff]
